# Atherosclerosis Imaging with ^18^F-Sodium Fluoride PET

**DOI:** 10.3390/diagnostics10100852

**Published:** 2020-10-20

**Authors:** Poul F. Høilund-Carlsen, Reza Piri, Caius Constantinescu, Kasper Karmark Iversen, Thomas J. Werner, Michael Sturek, Abass Alavi, Oke Gerke

**Affiliations:** 1 Department of Nuclear Medicine, Odense University Hospital, 5000 Odense, Denmark; Reza.Piri2@rsyd.dk (R.P.); Caius.Mihail.Constantinescu@rsyd.dk (C.C.); Oke.Gerke@rsyd.dk (O.G.); 2 Research Unit of Clinical Physiology and Nuclear Medicine, Department of Clinical Research, University of Southern Denmark, 5000 Odense, Denmark; 3 Department of Cardiology, Herlev Gentofte Hospital, 2900 Herlev, Denmark; Kasper.Karmark.Iversen@regionh.dk; 4 Department of Radiology, Perelman School of Medicine, University of Pennsylvania, Philadelphia, PA 19104, USA; Tom.Werner@pennmedicine.upenn.edu (T.J.W.); Abass.Alavi@pennmedicine.upenn.edu (A.A.); 5 Department of Anatomy, Cell Biology, Physiology, Indiana University School of Medicine, Indianapolis, IN 46202, USA; msturek@iupui.edu

**Keywords:** atherosclerosis, PET, ^18^F-sodium fluoride, NaF, calcification, quantification

## Abstract

The evidence on atherosclerosis imaging with ^18^F-sodium-fluoride (NaF) positron emission tomography (PET) is hotly debated because of the different patient characteristics, methodology, vascular beds, etc. in reported studies. This review is a continuation of a previous review on this topic, which covered the period 2010–2018. The purpose was to examine whether some of the most important questions that the previous review had left open had been elucidated by the most recent literature. Using principles of a systematic review, we ended analyzing 25 articles dealing with the carotids, coronary arteries, aorta, femoral, intracranial, renal, and penile arteries. The knowledge thus far can be summarized as follows: by targeting active arterial microcalcification, NaF uptake is considered a marker of early stage atherosclerosis, is age-dependent, and consistently associated with cardiovascular risk. Longitudinal studies on NaF uptake, conducted in the abdominal aorta only, showed unchanged uptake in postmenopausal women for nearly four years and varying uptake in prostate cancer patients over 1.5 years, despite constant or increasing calcium volume detected by computed tomography (CT). Thus, uncertainty remains about the transition from active arterial wall calcification marked by increased NaF uptake to less active or consolidated calcification detected by CT. The question of whether early-phase atherosclerosis and calcification can be modified remains also unanswered due to lack of intervention studies.

## 1. Introduction

A previous review of the literature on atherosclerosis imaging with ^18^F-sodium-fluoride (NaF) positron emission tomography (PET) published 2010–2018 left us with some major questions unanswered: (1) to what extent and with what consistency is arterial wall NaF uptake followed by or translated into CT-detectable calcification, (2) in which compartment of the arterial wall does the NaF-detectable microcalcification first occur, and (3) can NaF-avid microcalcification be diminished or abolished by medical therapy or other types of intervention [1]. To present the current state of the art, we have surveyed the most recent literature to examine if these important questions have been elucidated.

## 2. Materials and Methods

Using the same principles as previously described [1] including the Patient, Intervention, Comparison, Outcome Study (PICOS) approach [2], we searched PubMed/MEDLINE, Embase, and the Cochrane Library to extract relevant peer-reviewed articles on NaF-PET imaging in atherosclerosis published in English from 1 January 2019 and until 31 March 2020. We focused on (a) patients with any disease and (b) diagnostic performance, lesion detection, qualitative evaluation, and feasibility in (c) studies on evaluation of atherosclerosis. We had no restriction on comparator methods, outcome measures, or study design. Exclusion criteria comprised: (a) articles outside the scope of this review, (b) editorials, letters, comments, or conference proceedings, and (c) case studies, studies with other tracers than NaF, pure methodology studies, and review articles. One experienced researcher (PFHC) reviewed the full-text articles and extracted the following information: number, sex, age, and type of patients, tracer (NaF only or NaF and ^18^F-fluorodeoxyglucose (FDG)), artery segment studied, aim, quantification method, and main findings. What was reported in the literature is listed in Section 3 and debated in Section 4 using six subheadings (see below).

## 3. Results

We found 35 eligible articles. Three on the aortic valve (*n* = 3) and seven on methodology (*n* = 7) were excluded based on the abovementioned criteria, leaving 25 dealing with the coronary arteries [3,4,5,6,7,8,9,10,11], femoral artery [12,13,14,15], abdominal aorta [16,17,18], carotid arteries [19,20], thoracic aorta [21], and intracranial [22], renal [23], and penile arteries [24]. One study focused on the carotid and cerebral arteries [25], another on the carotids, the aortic arch, and coronary arteries [26], and a third on several major arteries [27]. Below (and in Supplementary Materials Tables S1–S5), the literature is summarized in six sections before being debated in the discussion:

- Disease mechanisms and targeting [10],
- Early detection and prevalence of NaF uptake in the heart and major arteries [5,23,24,25,27],
- NaF uptake in vulnerable, high risk, and ruptured plaque [6,7,8,9,19,20],
- Influence of age, sex, and other factors on NaF uptake [3,16,22],
- Association between NaF uptake and cardiovascular risk factors [4,15,21],
- NaF uptake and disease progression [11,12,13,14,17,18,26].

Mean or median differences presented below are statistically significant, except where otherwise stated.

### 3.1. Disease Mechanisms and Targeting

One experiment using an in vitro three-dimensional (3D) hydrogel collagen platform, ex vivo human coronary artery smooth muscle cells, excised carotid artery specimens, and a mouse model of atherosclerosis suggested that the NaF PET signal in PET-positive, CT-negative regions of human atherosclerosis plaques is the result of developing microcalcifications, and that high surface area in regions of small microcalcifications may amplify the PET signal [10].

### 3.2. Early Detection and Prevalence of NaF Uptake in the Heart and Various Arteries

Nakahara et al. found in 437 patients increased NaF uptake in the penile arteries of patients with erectile dysfunction present before (*n* = 336) or during treatment (*n* = 60) for prostate cancer (average maximal standardized uptake value (SUVmax) 1.88 and 1.86, respectively), while patients without erectile dysfunction (*n* = 41) had lower uptake (1.42) [24]. Dai et al. observed in 148 men and 31 women with various cancers and treatments that NaF uptake in multiple major arteries was slightly higher in plaques with the highest number of Hounsfield units. They portrayed the change in NaF uptake in plaques down through the arterial system from low in the carotid arteries via a peak in the abdominal aorta down to a modest level in the femoral arteries [27].

Fujimoto et al. found in 46 out of 54 carotid arteries from 28 patients scheduled for carotid endarterectomy or stenting due to ischemic vascular brain disease somewhat higher NaF than FDG uptake (mean target-to-background (TBR) 2.93 vs. 2.41) and increase in uptake of NaF but not FDG with more ischemic vascular brain disease according to magnetic resonance imaging [25]. Guaraldi et al. reported that coronary NaF uptake in HIV-positive patients without cardiovascular symptoms was higher than FDG uptake, but discordant with clinical risk assessment [5]. In what they termed “patients with NaF uptake,” Oliveira-Santos et al. reported (low) NaF uptake in the renal artery of six out of 25 patients with high cardiovascular risk hypertension and manifest cardiovascular symptoms. Those with NaF uptake were heavier, had higher triglycerides, high-sensitivity c-reactive proteins, and higher predicted cardiovascular risk, while their estimated glomerular filtration rate was lower and inversely correlated with renal artery NaF [23].

### 3.3. NaF Uptake in Vulnerable, High-Risk, and Ruptured Plaque

Hop et al. found in specimens of carotid plaques (17 vulnerable and six non-vulnerable) from 23 stroke patients undergoing carotid endarterectomy similar NaF uptake in culprit vs. non-culprit plaques and that only a median of 10% of CT-calcification volumes of interest (VOIs) showed increased NaF uptake, while a median of 35% of NaF PET VOIs showed calcification on CT. Moreover, in renal artery specimens from 15 healthy kidney donors they found no CT-calcification and much lower NaF uptake [19]. In contrast, Evans et al. calculated the maximal TBRmax in 26 patients with acute ischemic stroke and ipsilateral carotid stenosis and found that “most diseased segment TBRmax” was higher in culprit than non-culprit lesions for both NaF (2.68 vs. 2.39) and FDG (2.08 vs. 1.89), but independent of the degree of stenosis. Median NaF TBRmax was higher in bifurcations with high carotid artery calcium score, whereas the opposite pattern was seen with FDG; microcalcification by NaF-PET was a more focal process than inflammation by FDG-PET, which was more diffusely present [20].

Kwiecinski et al. reported in two publications NaF uptake in high-risk coronary plaques. In a study of 51 lesions in 23 patients (56%), increased NaF uptake was significantly associated with increased CT density of peri-coronary adipose tissue, which tends to co-localize with culprit lesions in patients with acute coronary syndromes [6]. In a study of patients screened for high-risk coronary plaques, 29 had plaques with positive remodeling, while 26 had low attenuation plaques. Two thirds of patients had positive coronary NaF uptake, and 70% of 68 high-risk lesions had increased NaF uptake. Of plaques with prognostically adverse features (positive remodeling, low attenuation plaque, spotty calcifications), low attenuation plaques had a sensitivity of 39% and a specificity of 98% for predicting NaF uptake; however, positive NaF uptake was often seen in the absence of low attenuation plaques. TBR was in general about 50% higher in lesions with low attenuation than in those without [7]. Kitagawa et al. demonstrated in 40 patients undergoing cardiac CT for suspected coronary artery disease that peri-lesional epicardial adipose tissue was positively associated with coronary TBRmax [8]. Finally, in an ex vivo study of two samples per nine autopsied patients and a total of 101 coronary plaques, Youn et al. demonstrated three times as high NaF uptake in plaques with compared to without microcalcification. Furthermore, higher NaF activity was associated with ‘advanced plaques’ characterized by fibroatheroma, but not with inflammation [9].

### 3.4. Influence of Age, Sex, and Other Factors on NaF Uptake

Raggi et al. found in 88 consecutive ambulatory patients without cardiovascular symptoms, 55 with type 2 diabetes (DM2), and 33 with type 1 diabetes (DM1) that coronary NaF uptake was increased in only 13 patients (15%). TBR was associated with male sex, estimated glomerular filtration rate, and total coronary artery score by CT, and TBR > median was associated with male sex and statin use [3]. Al-Zaghal et al. demonstrated positive correlations between NaF uptake and age in the choroid plexus and the epithalamus of healthy volunteers and that NaF uptake correlated with calcification in the same locations [22]. Arani et al. found in parts of the same material, positive correlations between age and 10-year Framingham Risk Score (FRS) and global abdominal aortic uptake of NaF, but not FDG, in 78 healthy volunteers and 45 patients [16].

### 3.5. Association between NaF Uptake and Cardiovascular Risk Factors

The study by Arani et al. demonstrated an association between NaF uptake and risk score, which was not present either for FDG [16]. Analyzing total heart NaF uptake, Sorci et al. made a comparison between 37 patients with angina pectoris and 37 healthy controls matched by age, gender, and BMI and found no differences between them with any calcium score, but patients had higher average SUVmean and FRS than controls; region of interest curves indicated that SUVmean could to some degree discriminate patients from controls, which FRS could not. In the entire material of 86 healthy controls and 50 angina pectoris patients, the authors observed that SUVmean increased with age and BMI, regardless of gender [4]. In 40 patients with suspected coronary artery disease, including 17 (43%) with DM, Ryoo et al. demonstrated that the burden of NaF in the descending thoracic aorta correlated significantly with DM and serum HbA1c level, whereas overall burden of CT-calcium deposition correlated with hypertension, metabolic syndrome, and 10-year CVD risk score [21]. Lastly, Takx et al. demonstrated that total cholesterol, triglycerides, and HbA1c are associated with NaF uptake in the femoral arteries of DM2 patients [15].

### 3.6. NaF Uptake and Disease Progression

Raggi et al. studied NaF uptake in 50 HIV-positive patients treated for > 6 months with anti-retroviral agents. All patients underwent cardiac CT 1–2 years apart and were examined by NaF-PET/CT at some time point after the second CT. In this cohort, of whom 66% (33) had hypertension and 28% (14) had DM2, 31 patients (62%) showed coronary artery calcification progression on CT. At least one territory with high NaF uptake was observed in 150 (50%) of 300 territories. High NaF uptake was present more often in areas classified by CT as non-calcified than calcified (68% vs. 32%). There was no relation between demographic and clinical variables including 10-year risk score on one hand and NaF uptake on the other. The NaF uptake did not correlate either with prior CT-assessed coronary artery calcification progression [26]. Chowdhury et al. studied 40 subjects imaged before and 6 months after percutaneous transluminal angioplasty of the femoral artery. In 14, who developed anatomic restenosis after 12 months, both baseline FDG and NaF uptake were significantly higher than in the 26 patients without restenosis. Uptake of both tracers increased from baseline to 6 months, whereas patients without restenosis experienced a decline in both tracers after 6 months. FDG coronary artery uptake above TBR 1.98 and NaF TBR above 2.11 were both highly discriminative with regard to restenosis after one year, whereas the index lesion CT calcium score was not. There was a significant positive correlation between symptomatic lesion uptake of NaF and calcium score, but not between FDG uptake and calcium score [12]. Zwackenberg et al. studied femoral artery NaF uptake in 35 women and men with DM2 and cardiovascular disease, who in a randomized trial received 360 µg/d menaquinone-7 (MK-7) or placebo. NaF uptake increased insignificantly in the MK-7 group, while a similar tendency was not observed with regard to CT-calcification. MK-7 treatment significantly reduced dephosphorylated-uncarboxylated matrix Gla protein. This study claimed that MK-7 was given to decrease atherosclerosis [13], but from a report by den Harder et al., it appears that the purpose was primarily to test the MK-7 effect on DM2 metabolic endpoints. The den Harder et al. study examined femoral NaF uptake in two cohorts, one of which apparently was part of the Zwackenberg cohort, while the other comprised a randomized trial on bisphosphonate (etidronate) treatment of pseudo-xanthoma elasticum. A higher TBR at baseline was associated with a higher femoral calcification mass at baseline and calcification progress in both studies. Analysis stratified per placebo or active drug showed the same direction and effect sizes in both cohorts. Slightly higher NaF TBR was observed at baseline in areas where CT-calcification was first visible at follow-up [14].

Cecelja et al. investigated the chronological change in NaF uptake in the abdominal aorta of 21 postmenopausal women. While NaF TBR remained constant after almost four years, there was a significant increase of 54% in abdominal aortic calcium volume [17]. Nakahara et al. observed in prostate cancer patients with at least three NaF PET/CT scans over >1.5 years that baseline NaF uptake was moderately correlated with age and BMI and tended to be higher in patients with hypertension (46% of cohort), whereas NaF uptake and calcium volume did not differ in patients with dyslipidemia, diabetes, history of coronary artery disease (CAD), or smoking. Within patients, NaF uptake varied from scan to scan, while CT calcium volumes remained constant or increased between scans. Nevertheless, NaF uptake correlated with calcium volume on the baseline scan and calcium volume increment. Patients with persistently high NaF uptake showed a higher calcium volume increment (0–1.5 years) than patients with low or transiently high NaF uptake. Their data also indicated absence of effect of statins on NaF uptake [18].

Finally, Moss et al. examined whether dual antiplatelet treatment with ticagrelor reduces high-sensitive troponin I concentration in patients with multi-vessel coronary disease, 94 randomized to ticagrelor and 97 to placebo. Triaged by NaF uptake in a proximal coronary artery, 71 presented a low uptake, while 120 had a high uptake (TBRmax > 1.25). There was no effect of ticagrelor at 30 days and one year in patients with increased coronary NaF uptake at baseline [11].

## 4. Discussion

The recent 25 articles reflect a growing interest in NaF PET/CT atherosclerosis imaging, compared to 16 papers in the preceding three-year period [1]. Studies varied in in size, purpose, and methodology; many were post-hoc analyzes performed for other purposes. Longitudinal studies that tell more about the connection between early phase NaF-avid arterial microcalcification and CT-detectable macrocalcification were in short supply.

### 4.1. Disease Mechanisms and Targeting

The experimental study by Creager at al. confirmed the prevailing perception that NaF targets microcalcifications that have a greater surface than aggregated calcification [10]. Regarding the question of which arterial wall compartment first experiences NaF-detectable microcalcifications, the Creager study demonstrated that NaF binds to microcalcifications formed by calcifying extracellular vesicles delivered from vascular smooth muscle cells, and it was shown in animal studies that exercise in hyperlipidemic mice (ApoE^−/−^) with baseline aortic calcification is associated with a reduction in aortic NaF uptake, whereas the fold change in aortic calcification measured by CT remained constant [28]. Moreover, when comparing uremic ApoE^−/−^ mice with non-uremic ApoE^−/−^ mice or control mice, the heart and close-by aorta showed both early and sustained NaF-uptake, which may be associated with endothelial activation in segments with vascular remodeling [29]. The extent to which these studies in rodent models translate to humans is not clear. Furthermore, as Hsu et al. [28] noted, it is possible that coronary artery calcification could be modulated very differently by exercise than aortic calcification. Large animal models will be essential for elucidating coronary artery calcification mechanisms in disease [30].

### 4.2. Early Detection and Prevalence of NaF Uptake in the Heart and Various Arteries

NaF uptake measured in the penile arteries of prostate cancer patients having undergone various treatments was more pronounced in patients with erectile dysfunction [24]. In stroke patients, where the ipsilateral carotid shows a higher NaF uptake, the increase correlates with the severity of the stroke [25]. Moreover, it was shown that rather few patients (15%) with high cardiovascular risk hypertension have slightly increased renal artery NaF uptake, but also that NaF uptake is associated with lower GFR and that estimated GFR is inversely correlated with renal artery NaF [23]. Finally, NaF uptake is increased in major arteries of patients with various cancers [27] and increased in HIV-positive patients and more so than FDG uptake [5].

### 4.3. NaF Uptake in Vulnerable, High-Risk, and Ruptured Plaque

The in vitro finding by Hop et al. [19] of similar NaF uptake in vulnerable and non-vulnerable carotid plaques from stroke patients questions the ability of increased NaF uptake to identify culprit lesions and underlines that NaF uptake may visualize a different stage of the calcification process than CT [31]. Evans et al. found in patients with acute ischemic stroke and ipsilateral carotid stenosis higher uptake of both NaF and FDG in culprit compared to non-culprit lesions and that NaF uptake was concentrated at the carotid bifurcation, while FDG uptake was evenly distributed throughout the arteries [20]. These discrepant findings could point to a critically variable transition between vulnerable (unstable) and stable calcified plaque as van Rosendael et al. found that very dense coronary artery calcification identified by CT decreases risk for acute coronary syndrome [32].

Examining ex vivo human coronary plaques, Youn et al. demonstrated three times as high NaF uptake in plaques with than without microcalcification and that higher NaF uptake was associated with intimal thickening, but not macrophage infiltration [9]. In patients with high-risk coronary plaques, Kwiecinski et al. [6,7] showed that increased NaF uptake in about half of lesions was associated with increased CT-density of peri-coronary adipose tissue, which seems to co-localize with culprit lesions in patients with acute coronary syndromes. Among adverse plaque features, low attenuation had a sensitivity of 39% and a specificity of 98% for predicting NaF uptake, but positive NaF uptake was often seen in the absence of low attenuation plaque [6,7]. Finally, Kitagawa et al. found that perilesional epicardial adipose tissue density correlated positively with cardiac NaF TBRmax in patients with known or suspected CVD, even if the volume of epicardial adipose tissue was similar in patients with coronary arterial uptake above and below their TBR cut-off [8].

### 4.4. Influence of Age, Sex, and Other Factors on NaF Uptake

Raggi et al. demonstrated positive coronary NaF uptake in only 15% of consecutive ambulatory middle-aged diabetics without cardiovascular symptoms, a finding that was associated with male sex, GFR, and CT total coronary calcium score [3]. It seems that the low rate of positive NaF uptake in these asymptomatic patients emphasizes that NaF may be a better marker of early-stage atherosclerosis than later developed CT-detectable plaque burden, which is a frequent finding in more developed diabetes [33]. This is consistent with the experimental findings of Moss et al., who demonstrated that NaF uptake tends to plateau in late stage atherosclerotic disease, when there is less calcified surface area to adsorb NaF [34]. Arani et al. found, in a reanalysis of scans from the so-called Cardiovascular Molecular Calcification Assessed by ^18^FNaF-PET/CT (CAMONA) cohort, a significant positive correlation between NaF uptake in the abdominal aorta and age in both healthy volunteers and angina pectoris patients, while a similar correlation was not present with FDG uptake [16]. Al-Zaghal et al. demonstrated in healthy volunteers from the same material significant positive correlations between NaF uptake in the right and left choroid plexus and the epithalamus and age, but no correlation of NaF uptake with Hounsfield units in the same locations [22].

### 4.5. Association between NaF Uptake and Cardiovascular Risk Factors

**Figure 1:**
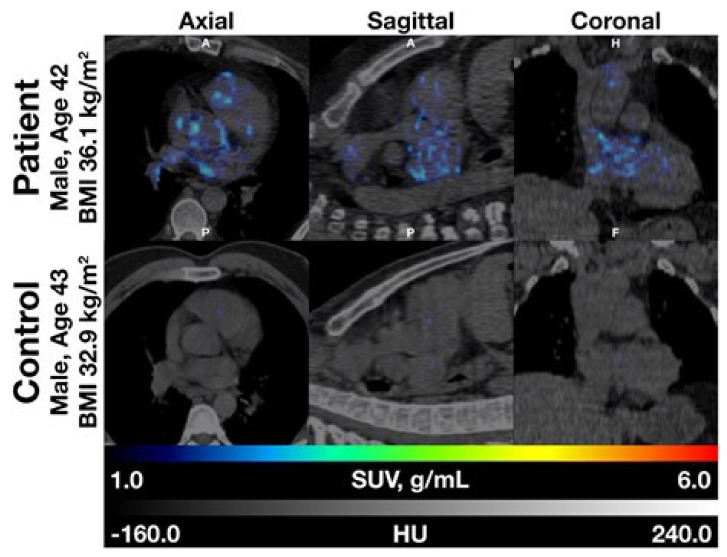
Fused NaF-positron emission tomography/computed tomography images of patient (top) and matched control (bottom). Note that the distribution of NaF uptake in the patient does not follow only the epicardial routes of the three coronary arteries, but is more dispersed, suggesting that NaF uptake is present in the walls of the entire cardiac arterial tree (with permission from reference [4]). Coronary arterial contrast-enhanced imaging would help address the issue.

Arani et al. found a positive correlation between NaF uptake, but not FDG uptake, in the abdominal aorta and 10-year FRS in both healthy volunteers and angina pectoris patients [16], and Sorci et al. observed that global heart NaF uptake and calcium scores were higher in patients than healthy volunteers (Figure 1) and that cardiac SUVmean could to some degree discriminate between patients and controls, whereas FRS could not. They also showed that cardiac SUVmean correlated with age and BMI across females and males and increased with age and BMI [4].

Ryoo reported that in patients with suspected CAD, the burden of NaF uptake in the descending thoracic aorta was significantly associated with diabetes mellitus and serum HbA1c level, while the overall burden of CT calcium deposition correlated significantly with hypertension, metabolic syndrome, and 10-year CVD risk score [21]. Takx et al. demonstrated that higher CT calcium, total cholesterol, and HbA1c were associated with higher NaF TBR in the femoral arteries of DM2 patients [15].

### 4.6. NaF Uptake and Disease Progression

Progression of CT-calcification was observed in almost two-thirds of HIV-positive patients following anti-retroviral treatment, when NaF uptake was present more often in non-calcified than calcified areas defined by CT [26]. Chowdhury et al. demonstrated that baseline uptake of both FDG and NaF could predict one-year femoral restenosis following percutaneous transluminal coronary angioplasty, whereas there was no difference in index lesion calcium score by CT between those who did or did not develop restenosis. There was a positive correlation between symptomatic lesion uptake of NaF, but not FDG, and calcium score [12]. Treatment of DM2 and cardiovascular disease with mena-quinone-7 (MK-7) was associated with an insignificant trend for increased NaF uptake in the femoral artery, whereas the same was not apparent for CT-calcification [13]. Furthermore, it was found that treatment with bisphosphonate (etidronate) in patients with pseudo-xanthoma elasticum was associated with a higher femoral calcification mass at baseline and calcification progress, and that slightly higher NaF TBR was present at baseline in areas with new CT-calcification at follow-up [14]. This seems to fit with the idea that NaF best measures the earliest stages of vascular calcification, while CT-calcification reflects late, high density, established calcification [7,10,32,33,35,36,37,38,39].

Noteworthy was the finding of unchanged NaF uptake into the abdominal aorta of postmenopausal women for almost four years, despite a significant (54%) increase in abdominal aortic calcium volume [17] and, similarly, of abdominal aorta NaF uptake that varied from scan to scan, while calcium volumes remained constant or increased between scans [18]. In the ticagrelor trial by Moss et al., coronary NaF uptake was used to stratify patients at baseline, but not to monitor the drug effect on coronary atherosclerosis [11].

### 4.7. Methodology

**Figure 2:**
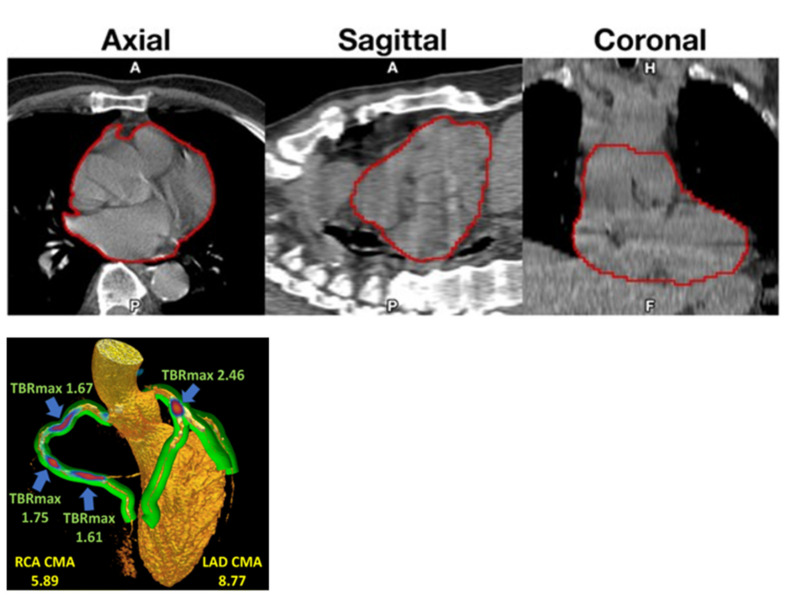
(**Top**): Volume of interest (VOI) capturing a representative heart. VOIs were thresholded to exclude voxels under −50 HU before being superimposed onto corresponding PET images (reproduced with permission from reference [4]). (**Left**): three-dimensional (3D) rendering of coronary CT angiography with superimposed tubular whole-vessel volumes of interest (light green) employed for evaluation of NaF uptake (blue and red). Despite the relatively lower TBRmax due to multiple foci of increased NaF activity, the coronary microcalcification activity (CMA) in the right coronary artery (RCA) is only moderately lower than in the left anterior descending (LAD) coronary artery, which presented with a very high TBRmax (with permission from reference [39]).

Standardization is lacking. Most centers calculated a TBR using SUVmax and/or SUVmean values divided by blood pool activity from various parts of the vascular bed, even if recorded activity varies considerably from segment to segment of the vascular bed dependent on spill-over from NaF content in adjacent bone [40]. Cardiac motion was addressed in particular by Kwiecinski et al., who measured the uptake in the proximal parts of the coronary arteries [41,42], which others have found to be suboptimal due to the limited spatial resolution of PET imaging and cardiac and respiratory movements [43]. Instead, a global assessment approach comprising the entire heart may be preferable [4,44], and perhaps a better reflection of cardiac atherosclerosis burden as a clinically more relevant measure than the NaF uptake in vulnerable coronary plaques [45,46]. Accordingly, Kwiecinki et al. proposed a novel “whole-vessel” coronary NaF approach for assessment of the “global coronary microcalcification burden” (Figure 2, left) [39]. It will be interesting to see how well this procedure corresponds with the whole-heart approach [4], which assumes that NaF is taken up only in the arterial walls and not in other tissues of the heart.

### 4.8. Limitations

**Figure 3:**
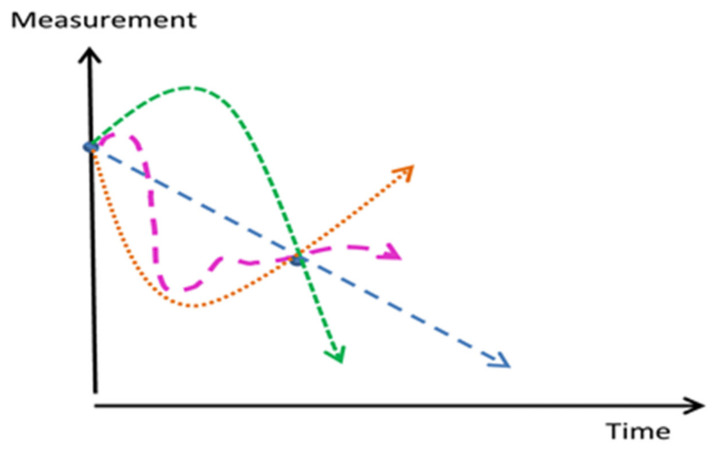
Most follow-up or intervention studies apply one baseline measurement and a single follow-up determination. If the follow-up point is lower than the baseline point, this is usually interpreted as a decline, although in reality one cannot know whether it represents a measurement on a different course, which may even be an opposite one. Three potential courses are indicated (dotted green, orange, and purple lines) (with permission from reference [47]).

The heterogeneity of studies and lack of quantification standardization hampered a direct comparison of studies. Most demonstrated relationships on vascular calcification were more indicative than actually predictive. Despite increasing scientific activity there remain large unexplored areas, which call for longitudinal trials applying at least 3 NaF-PET/CT scans over a long time period to characterize phases of calcification as shown in Figure 3 [47].

A relevant lesson from preclinical studies is that intracellular calcium stores during the progression of coronary atherosclerosis increase in early disease and decrease in late disease compared to healthy controls [48]. This biphasic relationship is illustrated in the green curve in Figure 3. Not a single prospective study has appeared that aims to show whether arterial NaF uptake, and potentially also later development of CT-detectable calcification, can be reduced. One reason might be the fear of inhibiting consolidation of existing CT-calcifications, which has been suggested as a negative, unintended outcome [32]. These high density (>1000 Hounsfield Units) nodules are proposed to be stable calcification sites, thus lowering risk for future acute coronary syndromes [32].

Only five studies enrolled more than 100 patients [4,11,16,24,27], and many were post-hoc analyses of patients examined primarily for other purposes, including cancer, which is somehow associated with cardiovascular disease [49,50]. The largest, with over 400 patients, was on penile arteries in patients with prostate cancer, most of which had erectile dysfunction [24], which may be caused by many other factors, including most cancer therapies, which severely affects the body, and thus, also the risk of developing atherosclerosis.

When it comes to association with cardiovascular risk factors including diabetes mellitus, none of the studies were tailored in a way that allowed conclusions about the extent to which NaF-PET/CT imaging can supplement or replace common risk assessment procedures. Thus, the study reporting surprisingly low coronary arterial NaF uptake in D1M or D2M patients [3] could not discriminate properly between categories and their respective treatments, nor could it explain the low NaF uptake in relation to previous studies demonstrating increased CT-detectable calcification in diabetic patients. Similarly, the study showing that the burden of NaF uptake in the descending thoracic aorta was significantly correlated with diabetes mellitus and serum HbA1c level, whereas the overall burden of CT calcium deposition was significantly correlated with hypertension, metabolic syndrome and 10-year CVD risk score, was not designed to further account for the shown statistical difference [21].

Thus, on the whole, a pervasive heterogeneity in terms of design, methods, patients, pre- or post-hoc analyzes, and the lack of prospective and longitudinal studies, focusing primarily on arteriosclerosis, hampered comparison of studies and extraction of reliable conclusions about their potential clinical impact.

### 4.9. Summary of Information Gleaned from the Literature

Listed below is what we think is now known about NaF PET imaging of atherosclerosis.

- Increased NaF uptake is seen in penile arteries of prostate cancer patients with erectile dysfunction and in the carotid artery ipsilateral to a recent stroke, but may be relatively rare in the renal arteries of high-risk hypertensive patients without cardiovascular symptoms. Slight increases are observed in many cancers and in HIV-positive patients.
- Increased NaF uptake in vulnerable coronary and carotid plaques can characterize these further, but not serve as a single identifying parameter because of the progression from molecular and “spotty” calcification that is an established marker for vulnerable plaque to high density calcification that may confer plaque stability and decreased risk of acute coronary syndromes.
- NaF uptake is positively associated with age and several other factors, but with a wide scatter calling for individual patient assessment.
- NaF is almost consistently associated with CV risk factors, but to what degree assessment of NaF uptake can substitute or enhance CV risk stratification remains unclear.
- Abdominal aorta NaF uptake appears not to progress significantly over a few years despite unchanged or increasing CT-calcification; this calls for studies of other arterial segments recognizing that there could be a steady-state conversion of NaF uptake (mainly micro- calcification) to high density macrocalcification measured by CT [34].
- It is not clear in which compartment of the arterial wall NaF-uptake first occurs; however, the weight of evidence indicates vascular smooth cells in the medial-intimal border [51].
- It is unknown if intervention can prevent or reduce development of arterial microcalcification and later appearance of CT-calcification. Mouse experiments seem to indicate that exercise increases consolidation and density of calcification sites, thereby decreasing surface area and risk of rupture in aorta. Additional experiments in coronary arteries are needed to assess more relevant risks for morbidity and mortality.

## 5. Conclusions

The literature on NaF-PET imaging of atherosclerosis is heterogeneous and diverging with regard to scope, design, size, methodology, and studied arterial segment, and devoid of proper anti-atherosclerotic intervention trials. A puzzling finding, demonstrated solely in the abdominal aorta, is the lack of progression of arterial wall NaF uptake over a period of 1.5–4 years despite constant or increasing CT-calcification. This raises questions regarding the value of NaF-PET/CT imaging in atherosclerosis, including whether NaF can be used to assess the efficacy of anti-arteriosclerotic therapy, perhaps except for the earliest stages of atherosclerosis. This calls for early diagnosis and clinical trials. This also calls for more targeted studies using safe and more standardized quantification methods.

## Supplementary Materials

The following are available online at https://www.mdpi.com/2075-4418/10/10/852/s1, Table S1: Jan 2019–Mar 2020 Studies on Early Detection and/or Prevalence, Table S2: Jan 2019–Mar 2020 Studies on NaF Uptake in Vulnerable, High-Risk, and Ruptured Plaque, Table S3: Jan 2019–Mar 2020 Studies on Influence of Age, Sex, and Other Factors on NaF Uptake, Table S4: Jan 2019–Mar 2020 Studies on Arterial NaF Uptake and Cardiovascular Risk Factors, Table S5: Jan 2019–Mar 2020 Studies on Progression and Intervention.
