# Supplementary material for: Atherosclerosis Imaging with 18F-Sodium Fluoride PET
Source: Diagnostics (Basel). 2020 Oct 20;10(10):852. doi: 10.3390/diagnostics10100852 (PMC7590213; doi:10.3390/diagnostics10100852)
Supplement: Supplementary file 1 [file diagnostics-10-00852-s001.pdf]

Atherosclerosis Imaging with ^{18}F-Sodium Fluoride PET

Supplementary Materials

Contents

Table S1: Jan 2019–Mar 2020 Studies on Early Detection and/or Prevalence 2

Table S2: Jan 2019–Mar 2020 Studies on NaF Uptake in Vulnerable, High-Risk and Ruptured Plaque 4

Table S3: Jan 2019–Mar 2020 Studies on Influence of Age, Sex and Other Factors on NaF Uptake 6

Table S4: Jan 2019–Mar 2020 Studies on Arterial NaF Uptake and Cardiovascular Risk Factors 7

Table S5: Jan 2019–Mar 2020 Studies on Progression and Intervention 8

Table S1. Jan 2019–Mar 2020 Studies on Early Detection and/or Prevalence.

First Author (Ref. #) Affiliation	Patients, *n* (females) Age in years Mean ± SD or range	Material	Tracer	Arterial Segment	Purpose	Quantification	Main Findings	Comment
Nakahara et al. Apr, 2019 [24] Mount Sinai, New York, USA	437 (0) 66.6 ± 8.7 y	Prostate cancer pts	NaF	Penile arteries.	Penile artery atherosclerosis and erectile dysfunction.	Average SUVmax.	3 groups with varying degree of erectile dysfunction (ED): Prevalent ED (*n* = 336); Incident ED (*n* = 60); No ED (*n* = 41). Pts with prevalent and incident ED had similar NaF uptake (median 1.88 and 1.86) while no ED pts had significantly lower uptake (1.42). After adjustment for other risk factors the odds ratio of prevalent or incident ED was 25.2 for every 0.5-U increment in SUVmax with ROC area of 0.91.	
Dai et al. Jun 2019 [27] Houston, US, Tianjin, China	179 (31): 122 aged 65.7±8.9 y with NaF uptake or calcium + 57 aged 47.1 ± 20.6 y without.	Consecutive pts: 148 men and 31 women with various cancers.	NaF	Common carotids, brachiocephalic trunk, aortic arch, thoracic and abdominal aorta, iliac and femoral arteries.	Association of arterial NaF uptake with CT calcification.	TBR = SUVmax of arterial plaque/SUVmean of blood pool in lumen of artery. Arterial plaque NaF positive if TBR >1.5.	Note: 148 men with prostate (110), thyroid (17), osteosarcoma (12), neuroendocrine (2) and other cancers (7) and 31 women with thyroid (13), breast (11) and other cancers (7). Three categories: TBRlow ≤1; TBRmed 1– ≤ 1.5; TBRhigh > 1.5. Plaques with either positive NaF uptake or a calcium score (CS) on CT were observed in 122/179 pts (76%). NaF uptake judged from TBR was slightly higher in plaques with the highest Hounsfield units, but the difference in uptake btw 4 calcification categories was small. There was a slight, but significant positive correlation btw NaF uptake and CT calcification.	
Fujimoto et al. Dec 2019 [25] Kagawa, Japan	28 (1) 70 (53–82) y	Pts scheduled for carotis endarterectomy or stenting	NaF & FDG	Carotids & cerebral hemispheres.	Association btw carotid NaF and FDG uptake and severity of ischemic vascular brain disease on MRI	TBR = SUVmax of plaque/SUVmean of internal jugular vein activity.	Focal NaF and focal FDG uptake was noted in the same 46 of 54 carotids. 4 were NaF positive and FDG negative, 1 was NaF negative and FDG positive, and 3 were NaF negative and FDG negative. Mean NaF TBR was higher than mean FDG TBR: 2.93 vs. 2.41. NaF uptake higher with increasingly more ischemic vascular brain disease according to MRI; similar relationship was not noted for FDG.	
Guaraldi et al. Feb 2020 [5] Modena, IT,	(1) 50 (8) 57.1±7.8	(1) 50 adult HIV+ pts treated ≥6 mo with antiretroviral agents had 2 CTs	NaF	Coronaries.	Comparison of prevalence and amount of NaF vs. FDG uptake in	TBR = SUVmax (coronary) / SUVmean RA blood pool;	Among a total of 93 pts, 39 (42%) were considered at low (10 y) and 54 (48%) at high CV risk (AHA/ACC criteria). NaF coronary uptake in general somewhat (40-60%) higher than FDG uptake. Frequency of increased NaF and increased FDG uptake similar in low and high-risk patients.	Post hoc analysis.

**First Author (Ref. #) Affiliation**	**Patients, *n* (females) Age in years Mean ± SD or range**	**Material**	**Tracer**	**Arterial Segment**	**Purpose**	**Quantification**	**Main Findings**	**Comment**
Edmonton, Canada	(2) 43 (x)<br><br>Total:<br>93 (16)<br>57.1±7.1	1-2 y apart and 1 NaF PET/CT after #2 CT.<br>(2) 43 HIV+ pts with FDG PET/CT.<br>Both groups without symptomatic CVD.	FDG		coronary arteries of HIV+ pts.	TBR ≥ 1.6 was considered abnormal.	Waist circumference, CRP, D-dimer, HIV duration, and treatment with nucleoside reverse transcriptase inhibitors were associated with high NaF uptake in univariate analyses; so was D-dimer in multivariable analyses, whereas there were no significant associations with FDG uptake. Thus, results indicate discordance btw clinical and imaging-based risk assessment.	
Oliveira-Santos et al. Feb 2020 [23] Coimbra, Portugal	25 (14)<br>63.9±8.6 y	High CV risk hypertensive subjects without manifest CVD.	NaF	Renal artery.	NaF uptake in the renal atery in this particular type of subjects.	TBRmax = SUVmax of lesion/TBRmean of activity in vena cava superior blood.	Six individuals showed (low) renal artery wall NaF uptake (TBR 1.4±0.4). They were heavier, had higher triglycerides, high-sensitivity CPR and predicted CV risk (SCORE) thant the remaining cohort. Trend toward higher NaF uptake in other major vessels and increased thoracic fat volume in subjects with renal artery NaF uptake. GFR was lower in subjects with NaF positive plaques and renal artery NaF uptake and eGFR were inversely correlated.	

CAD = coronary artery disease; CV = cardiovascular; CVD = cardiovascular disease; DM = diabetes mellitus; FDG = ^{18}F-fluorodeoxyglucose; NaF = ^{18}F-sodium fluoride; pts = patients; SQS = subjective quality score; SUV = standardized uptake value; TBR = target-to-background ratio; Y = years.

Table 2. Jan 2019–Mar 2020 Studies on NaF Uptake in Vulnerable, High-Risk, and Ruptured Plaque.

First Author (Ref. #)	Patients, *n* (females) Age in years Mean±SD or range	Material	Tracer	Arterial Segment	Purpose	Quantification	Main Findings	Comments
Hop et al. Aug 2019 [19] Groningen, NL	23 (4) 72 (55–76) y<br>15 (10) 55 (41–63) y	Carotid plaques (17 vulnerable + 6 non-vulnerable) from 23 stroke pts with carotid endarterectomy.<br>15 renal arteries from healthy kidney donors.	NaF	Carotids.	Comparison of NaF uptake in culprit and non-culprit lesons and with CT calcification.	NaF uptake in VOIs corrected for specimen weight and incubation dose: % uptake of total incubation dose per gram tissue (%Inc/g).	NaF uptake was similar in culprit vs. non-culprit plaques (2.32 vs. 2.35 %Inc/g).<br>Only a median of 10% of CT calcification VOI showed increased NaF uptake.<br>Only a median of 35% of NaF PET VOI showed calcification on CT.<br>Renal arteries were without CT calcification and had much lower NaF uptake.	In vitro study. Questions the ability of increased NaF uptake to identify cultprit lesions.<br>NaF uptake appears to visualize a different stage of the calcification process than CT.
Kwiecinski et al. Oct 2019 [6] Los Angeles, Edinburgh, Seoul	41 (13) 65±6 y	Pts with high-risk coronary plaques (HRPs) according to cardiac CT.	NaF	Coronaries	Relationship btw increased lesion peri-coronary adipose tissue (PCAT) and NaF uptake in pts with stable HRPs.	Special motion correction, TBR = SUVmax of regions with a >25% stenosis/RA blood pool activity; TBR > 1.25 was considered positive.	Background: Pathologic studies have identified a large lipid core, spotty calcifications, positive remodeling, and inflammatory cell infiltration as "adverse features" of ruptured coronary plaques. However, the positive predictive value for myocardial infarction has been shown to be low.<br>Here, 51 lesions in 23 pts (56%) showed increased NaF uptake significantly associated with increased CT density of peri-coronary adipose tissue (PCAT), (seems to co-localize with culprit lesions in pts with acute coronary syndromes) and with low attenuation plaque volume.	Post hoc analysis.
Kwiecinski et al. Jan 2020 [7] Los Angeles, Warsawa, Seoul, Edinburgh.	55 (22) 67 (57–71) y	Pts at two centers screened for high-risk coronary plaques (cf. study above) and split into 29 (17) with positive remodeling and 26 (10) with low attenuation plaque.	NaF	Coronaries	Search for qualitative and quantitative cardiac CT predictors of abnormal coronary NAF uptake.	As above.	Of 55 pts, 35 (64%) had positive coronary NaF uptake. Of 68 high-risk lesions, 49 (70%) had increased NaF uptake. Of so-called prognostically important adverse plaque features (positive remodeling, low attenuation plaque, spotty calcifications) low attenuation plaque a had sensitivity 39% and specificity 98% for 'predicting' NaF uptake, but positive NaF uptake was often seen in the absence of low attenuation plaque. TBR was in general about 50% higher in lesions with low attenuation then those without.	Post hoc analysis.
Kitagawa et al. Jan 2020 [8]	40 (8) 68 ± 7 y	Pts undergoing cardiac CT for known or suspected CVD.	NaF	Coronaries	Relationship btw coronary NaF uptake and	TBRmax = lesion SUVmax/vena cava superior	EAT volume was similar in pts with TBR above and below 1.28, but perilesional EAT density was positively correlated with TBRmax.	

First Author (Ref. #)	Patients, *n* (females) Age in years Mean±SD or range	Material	Tracer	Arterial Segment	Purpose	Quantification	Main Findings	Comments
Hiroshima, Japan					epicardial adipose tissue (EAT) by CT.	SUVmean. TBR ≥ 1.28 ~ positive.		
Youn et al. Feb 2020 [9] New York, USA	9 pts autopsied 2015-16 Age and sex not stated	Ex vivo human coronary arteries, two samples per patient, 101 plaques in total.	NaF	Coronaries	Correlation btw NaF uptake and micro- and macrocalcification in ex vivo human coronary plaques.	TBR = max counts in region in region of interest divided by max counts in the region "without significant" NaF activity.	Three times as high NaF uptake in plaques with microcalcification than in plaques without. Higher NaF activity was associated with 'advanced plaques' characterized by fibroatheroma (3 times higher than in plaques with intimal thickening and twice as high as in plaques with pathological intimal thickening). No significant association btw NaF activity and inflammation (number of macrophages assessed by immuno-histochemical staining for CD68).	Post mortem study.
Evans et al. Mar 2020 [20] Cambridge, UK	26 (8) 74.8 ± 9.7 y	Pts with acute ischemic stroke with ipsilateral carotid stenosis of ≥50%.	NaF & FDG	Carotids	NaF and FDG uptake in culprit vs. non-culprit lesions, spatial distribution of uptake, and how macrocalcification affects.	TBRmax = SUVmax/SUVmean of jugular vein blood pool activity.	Median most diseased segment TBRmax was higher in culprit than non-culprit lesions for both NaF (2.68 vs. 2.39) and FDG (2.08 vs. 1.89). The uptake of both was independent of the degree of stenosis. Whole vessel TBRmax in culprit lesions was slightly, but significantly, higher for FDG (1.92 vs. 1.71, $p < 0.001$), and insignificantly so for NaF (1.85 vs. 1.79, $p = 0.10$). NaF uptake was concentrated at carotid bifurcations, while FDG was distributed evenly throughout arteries. Weak correlation btw NaF and FDG uptake across all carotid bifurcations, but median NaF TBRmax was higher in bifurcations with high than low carotid artery calcium score (CACS) whereas the opposite pattern was seen with FDG.	Microcalcification (increased NaF uptake) seems to be a more focal process mainly located at the bifurcation (and potentially in response to biomechanical factors), while inflammation (increased FDG uptake) is a more diffuse process (due to what?).

CAD = coronary artery disease; CV = cardiovascular; CVD = cardiovascular disease; DM = diabetes mellitus; FDG = 18F-fluorodeoxyglucose; NaF = 18F-sodium fluoride; pts = patients; SQS = subjective quality score; SUV = standardized uptake value; TBR = target-to-background ratio; Y = years.

Table 3. Jan 2019–Mar 2020 Studies on Influence of Age, Aex and Other Factors on NaF Uptake.

First Author (Ref. #)	Patients, *n* (females) Age in years Mean±SD or range	Material Pro- or Retrospective	Tracer	Arterial Segment	Purpose	Quantification	Main Findings	Comments
Raggi et al. Feb 2019 [3] Edmonton Canada	88 (31) 54.0 ± 14.0	Consecutive ambulatory pts with DM, 55 DM2 + 33 DM1 – all asymptomatic for CVD. Prospective.	NaF	Coronary arteries.	Prevalence of increased NaF uptake in what because of that was considered potentially vulnerable plaques.	TBR = SUVmax (coronary)/SUVmean LV blood pool; TBR ≥ 1.5 was considered abnormal.	TBR ≥ 1.5 in 13 pts (15%). TBR associated with male sex, estimated GFR, and total coronary artery score by CT. TBR > median associated with male sex and statin use. No follow-up data.	
Al-Zaghal et al. Feb 2019 [22] Philadelphia, US, Odense, Denmark	78 (38) 45.3 ± 14.2 y	Healthy control subjects.	NaF	Choroid plexus and epithalamus.	Feasibility of NaF PET/CT to assess calcification of intracranial structures.	SUVmean of VOIs.	Significant positive correlations btw NaF uptake and age in the right and left choroid plexus and the epithalamus. NaF uptake in these three locations correlated also with HUs in the same locations.	
Arani et al. 2019 [16] Philadelphia, US + Odense, Denmark	123 (61) 48 ± 14 y	78 health volunteers + 45 angina pts. Reanalysis of prospectively collected material.	NaF + FDG	Abdominal aorta.	Correlation of uptake with age and risk (FRS, see Table 4).	Global tracer uptake value score (SUVmean x volume).	Significant positive correlation between both age and 10 years' FRS and global abdominal aorta uptake of NaF, but not FDG, in both volunteers and patients.	One of few studies on the abdominal aorta.

CAD = coronary artery disease; CV = cardiovascular; CVD = cardiovascular disease; DM = diabetes mellitus; FDG = 18F-fluorodeoxyglucose; NaF = 18F-sodium fluoride; pts = patients; SQS = subjective quality score; SUV = standardized uptake value; TBR = target-to-background ratio; Y = years.

Table S4. Jan 2019–Mar 2020 Studies on Arterial NaF Uptake and Cardiovascular Risk Factors.

First Author (Ref. #)	Patients, *n* (females) Age in years Mean±SD or range	Material	Tracer	Arterial Segment	Purpose	Quantification	Main Findings	Comments
Sorci et al. Nov 2019 [4] Philadelphia, US + Odense, Denmark	136 ( ) 21–75 y	86 healthy controls and 50 angina pectoris patients.	NaF	Heart.	NaF PET/CT vs. calcium and Framingham scoring for preventive CAD intervention	SUVmean and SUVmax from whole heart minus superior vena cava blood pool activity.	Intergroup comparisons were made in 37 patient/control pairs matched by age, gender, and BMI. Calcium scores and SUVmax did not differ between pts and controls, but pts had higher average SUVmean ($p = 0.006$) and Framingham scores ($p = 0.02$) than controls. However, ROC curves indicated that SUVmean could "discriminate" pts from controls (AUC = 0.63, $p = 0.049$), whereas Framingham scores could not (AUC = 0.44, $p = 0.38$). SUVmean correlated with age and BMI among females and males. SUVmean increased with age and BMI.	
Ryoo et al. Jan 2020 [21] Seoul, Korea	40 (7) 62.1 ± 8.5 y	Pts with suspected CAD.	NaF	Descending thoracic aorta.	Correlation btw NaF uptake and CV risk factors	Average TBRmax = summed TBRmax of all slices / SUVmean of superior vena cava blood pool.	The burden of NaF uptake was significantly correlated with diabetes mellitus and serum HbA1c level. The overall burden of CT calcium deposition was significantly correlated with hypertension, metabolic syndrome and 10-year CVD risk score.	
Takx et al. March 2020 [15] Utrecht and Amsterdam, NL	68 (16) 69 ± 8 y	Subjects with DM2 and known arterial disease.	NaF	Femoral.	Potential determinants of NaF uptake in these specific pts.	TBR = SUVmax of femoral ROI / SUVmean of the blood pool activity in superior vena cava.	After correction for age and sex, higher CT calcium, total cholesterol, and HbA1c were associated with higher NaF TBR.	

CAD = coronary artery disease; CV = cardiovascular; CVD = cardiovascular disease; DM = diabetes mellitus; FDG = 18F-fluorodeoxyglucose; NaF = 18F-sodium fluoride; pts = patients; SQS = subjective quality score; SUV = standardized uptake value; TBR = target-to-background ratio; Y = years.

Table S5. Jan 2019–Mar 2020 Studies on Progression and Intervention.

First Author (Ref. #)	Patients, *n* (females) Age in years Mean ± SD or range	Material	Tracer	Arterial Segment	Purpose	Quantification	Main Findings	Comments
Raggi et al. Mar 2019 [26] Edmonton Canada & Modena, Italy	50 (8) 57.1 ± 7.8	HIV+ pts treated for ≥ 6 mo with anti-retroviral agents, who had 2 cardiac CTs 1–2 years apart were examined once with NaF PET/CT at an unknown time point after CT #2.	NaF	Carotids, aortic arch, coronaries.	Relationship btw prior CAC progression and current NaF activity in several arterial beds.	As in Raggi et al. (4) for coronaries and as SUVmax/ SUVmean superior vena cava blood pool for aortic arch and carotids, TBR ≥ 1.6 was considered abnormal.	Heterogeneous material with various treatments and including 66% with hypertension and 28% with DM2. 31 pts showed CAC progression by CT and 19 did not. At least one territory with high NaF uptake was observed in 150 (50%) of 300 territories. High NaF uptake more often in non-calcified than calcified areas (68% vs. 32%). No relation btw demographic and clinical variables including 10 y risk score. NaF uptake did not correlate with prior CT-assessed CAC progression.	Remarkably high frequency of increased NaF uptake, but unknown interval btw NaF PET/CT and last coronary CT.
Moss et al. Aug 2019 [11] Edinburgh, UK	191 (39) 65.9 ± 8.3	Patient with multivessel coronary artery disease.	NaF	Proximal coronaries.	Effect of dual antiplatelet therapy with ticagrelor on troponin I concentration.	TBRmax = SUVmax in proximal artery plaque divide by activity in plaque without traceable NaF uptake.	Unchanged troponin I concentration at 30 days and one year in patients (*n* = 121) with increase NaF uptake in a proximal coronary artery defined as TBRmax >1.25.	NaF uptake used for stratification at baseline, not for monitoring therapy.
Cecelja et al. May, 2019 [17] King's College London, UK	21 (21) 62 ± 6 y	Postmenopausal women with prior NaF PET/CT for assessment of bone mineralization.	NaF	Abdominal aorta.	NaF uptake as indicator of calcification progression (judged by CT).	TBRmean and TBRmax as ratios of SUVmean and SUVmax in aorta and SUVmean in vena cava.	No change in NaF TBR after 3.8 ± 1.3 years of follow-up despite a significant increase in abdominal aortic calcium volume (0.46 to 0.71 cm^3).	Longest before–after interval. No change in NaF TBR despite increase in calcification volume.
Chowdhury at al. Jun 2019 [12] Cambridge and Edinburgh, UK, Harvard, US, Copenhagen, DK	40 (14) 71.5 (65–79) y	Subjects imaged before and 6 mo after percutaneous transluminal angioplasty (PTA) of the superficial femoral artery.	NaF & FDG	Femoral artery.	Prediction of restenosis following PTA.	TBRmax = maximal femoral SUV divided by SUVmean of femoral vein.	In 14 pts who developed anatomic restenosis after 12 mo both baseline FDG and baseline NaF uptakes were significantly higher than in the 26 pts who did not develop restenosis and uptake of both tracers increased from baseline to 6 mo, whereas the lower uptake of both tracers in those without restenosis had declined after 6 mo. A cutoff of FDG TBR = 1.98 and NaF TBR = 2.11 (at baseline?) was highly discriminative with regard to restenosis after 1 year whereas there was no difference in index lesion calcium score by CT btw those who did or not did develop restenosis. There was a significant positive correlation btw symptomatic lesion uptake of NaF and calcium score, but not btw FDG uptake and calcium score.	Interesting new finding worth investigating in new and larger prospective studies.
Zwackenberg et al. Oct 2019 [13] Utrecht, Groningen, etc., NL	35 (9 f) 69.1 ± 8.4 y vs 33 (7 f) 69.1±8.4 y	Men and women with type 2 diabetes and CVD receiving 360 µg/d menaquinone-7 (MK-7) or placebo.	NaF	Femoral arteries.	Placebo-controlled RCT.	NaF uptake (90 min acquisition) measured as TBR (femoral SUVmax / vena cava SUVmean)	Femoral artery (from to) uptake of NaF (90 min acquisition) measured as TBR (femoral SUVmax / vena cava SUVmean) was primary and calcification mass by CT was secondary outcome before and following 6 mo of treatment. TBR tended to increase (insignificantly) in the MK-7 group while a similar tendency was not observed with regard to CT calcification. MK-7 treatment significantly reducesd dephosphorylated-uncarboxylated matrix Gla protein (as it should).	First intervention study. Vitamin K supplementation tended to increase femoral wall uptake of NaF compared to placebo and did not reduce CT calcification.

First Author (Ref. #)	Patients, *n* (females) Age in years Mean ± SD or range	Material	Tracer	Arterial Segment	Purpose	Quantification	Main Findings	Comments
Nakahara et al. Nov 2019 [18] New York, US and Tokio, Japan	45 (45) 67.0±9.2 y	Prostate cancer pts with at least 3 NaF PET/CTs over at least 1.5 years.	NaF	Abdominal aorta.	Relationship btw NaF uptake cnd CT evident calcification.	SUVmax of each slice was summed and devided by the number of slices to provide an average SUVmax/slice.<br>No TBR.	The median SUVmax of all patient scans were used to divide pts into three groups depending on the NaF uptake:<br>Persistently low (all scans below the median)<br>Transiently high (<50% of scans: SUVmax/slice > median)<br>Persistently high (>50% of scans: SUVmax/slice > median).<br>At baseline NaF uptake was moderately correlated with age and BMI and was higher in pts with hypertension. NaF uptake and calcium volume did not differ in pts with dyslipidemia, diabetes, history of CAD or past smoking. No effect of warfarin, slight effect from statins. NaF uptake varied from scan to scan while calcium volumes remained constant or increased between scans**.** NaF uptake correlated with calcium volume on baseline scan and calcium volume increment, especially from 1 to 1.5 years. Pts with persistently high NaF uptake showed higher calcium volume increment (0–1.5 years) than pts with low or transiently high NaF uptake.	29 pts had hypertension<br>30 pts had dyslipidemia<br>12 pts had diabetes<br>11 pts had a history of CAD<br>28 pts were current/past smokers<br>23 pts had statins at initial or follow-up scan<br>2 pts started warfarin during follow-up.<br><br>NaF uptake waxed and waned between scans.
den Harder et al. Jan 2020 [14] Utrecht, Amsterdam, Groningen, NL	TEMP: 36(17)+36(17) pts aged 56.7±8.6 & 57.3±8.1 y<br><br>VITACAL: 30(5)+26(7) pts aged 68.2±7.5 & 70.8±7.8 Y	From 2 RCTs:<br>TEMP: 1 yr treatment with bisphosphonate (etidronate) and placebo of pts with pseudo-xanthoma elasticum<br>VITICAL: pts with DM2 receiving 360 µg vitamin K and placebo in 6 mo.	NaF	Femoral arteries.	Can arterial NaF activity assess presence and predict progression of CT calcification?	TBR = SUVmax of femoral ROI / SUVmean of the blood pool activity in superior vena cava.	NaF PET/CT at baseline and after 12 mo or 6 mo follow-up.<br>A higher TBR at baseline was associated with a higher femoral calcification mass at baseline and calcification progress in both studies.<br>Analysis stratified per group (placebo or active drug) showed the same direction and effect sizes in both materials.<br>Slightly higher NaF TBR was observed at baseline in areas with new CT calcification at follow-up.	Complicated study with very differing materials, but nonetheless very similar results. Results of separate analysis of the two placebo groups were not presented in the article text.

CAD = coronary artery disease; CV = cardiovascular; CVD = cardiovascular disease; DM = diabetes mellitus; FDG = ^{18}F-fluorodeoxyglucose; NaF = ^{18}F-sodium fluoride; pts = patients; SQS = subjective quality score; SUV = standardized uptake value; TBR = target-to-background ratio; Y = years.
